# The decade of Roma Inclusion: did it make a difference to health and use of health care services?

**DOI:** 10.1007/s00038-017-0954-9

**Published:** 2017-03-29

**Authors:** János Sándor, Zsigmond Kósa, Klára Boruzs, Julianna Boros, Ildikó Tokaji, Martin McKee, Róza Ádány

**Affiliations:** 1 Department of Preventive Medicine, Faculty of Public Health, University of Debrecen, Kassai str 26/B, 4028 Debrecen, Hungary; 2 Department of Preventive Medicine, Faculty of Public Health, WHO Collaborating Centre on Vulnerability and Health, University of Debrecen, Debrecen, Hungary; 3 Department of Methodology for Health Visitors and Public Health, Faculty of Health, University of Debrecen, Nyíregyháza, Hungary; 4 Department of Health Systems Management and Quality Management in Health Care, Faculty of Public Health, University of Debrecen, Debrecen, Hungary; 5 Central Statistical Office, Budapest, Hungary; 6 ECOHOST, London School of Hygiene and Tropical Medicine, London, UK; 7 MTA-DE Public Health Research Group, University of Debrecen, Debrecen, Hungary

**Keywords:** Roma, Decade of Roma Inclusion, Health behaviour survey, Public policy

## Abstract

**Objectives:**

We investigated whether the severely disadvantaged health of Hungarian Roma adults living in segregated settlements changed by the Decade of Roma Inclusion program.

**Methods:**

We compared the results of two paired health interview surveys that we carried out using the same methodology before and after the Decade, on the general Hungarian and Roma populations.

**Results:**

Self-perceived health status of younger Roma worsened, while it improved among older Roma. Reported experience of discrimination reduced considerably and health care utilization improved in general. Positive changes in smoking and nutrition, and negative changes in alcohol consumption and overweight were observed. Many of observed changes can plausibly be linked to various government policies, including a quadrupling of public works expenditure, banning smoking in public places, restricting marketing of tobacco products, increasing cigarette prices, and a new tax on unhealthy foods. Liberalization of rules on alcohol distillation coincided with worsening alcohol consumption.

**Conclusions:**

We have shown that Roma remain severely disadvantaged and present an innovative sampling method which can be used to monitor changes in groups where identification is a challenge.

**Electronic supplementary material:**

The online version of this article (doi:10.1007/s00038-017-0954-9) contains supplementary material, which is available to authorized users.

## Introduction

Roma are the largest ethnic minority in Europe, with estimates of their numbers ranging from 10 to 12 million, most living in Central and Eastern Europe (CEE) (European Commission 2011). Historically, they have suffered severe disadvantage. Although many have assimilated with majority populations, large numbers continue to live on the margins of society, in segregated settlements with poor housing and few amenities, excluded from mainstream education and the labour market. Many studies have noted their poor health and high levels of unmet health need and impaired access to care (Arora et al. 2016; Cook et al. 2013; Hajioff and McKee 2000).

Their conditions rose high on the political agenda when the countries of CEE countries began accession to the European Union (EU), which required commitments to improve their rights. Soon after accession, international organisations, governments, and civil society came together in 2005 in the Decade of Roma inclusion (2015). This sought progress in targeted sectors, i.e. housing, education, employment and health. Subsequent European policies have also addressed this issue, including the EU’s Europe 2020 program, as have national initiatives.

Whether these initiatives have actually improved the situation of Roma is not, however, clear, despite the importance of knowing what did or did not work (Cook et al. 2013; Decade of Roma Inclusion 2015; Rorke et al. 2015). For example, progress towards the sustainable development goals (SDGs), requiring improvements in the social, economic, and environmental conditions of the entire population. Moreover, faced with aging populations, the EU cannot accept that several million citizens remain excluded from the labour force (Fésüs et al. 2012; Koupilová et al. 2001; Ringold et al. 2005).

Although some indicators were specified within the framework of the Decade (Kushen 2015), most routine information systems in the region largely lack ethnicity data. Moreover, there are methodological challenges relating to identification as Roma, reflected in the varying estimates of their numbers derived from different sources, itself a reflection of degrees of assimilation and discrimination. In some countries, many Roma lack documentation establishing their citizenship. Those surveys that have been undertaken, most notably a multi-country study conducted by UNDP in 12 countries in 2011, have sampled Roma living in identifiable settlements, typically separate from, but in close proximity to the majority population (Kühlbrandt et al. 2014).

In this study, we take advantage of two unique surveys, undertaken using the same methodology, and designed to be comparable with national surveys of the general Hungarian population, of Roma living in such settlements in Hungary, assessing changes between 2004 and 2015, thereby spanning the entire Decade of Roma Inclusion. Thus, we contribute to the sparse literature on progress achieved during the Decade of Roma Inclusion.

## Methods

The surveys of the general Hungarian population were undertaken in 2003 and in 2014. Details of the 2003 survey have been published elsewhere (Kósa et al. 2007) and of the 2014 survey, which used the same approach, are available from the Hungarian Central Statistical Office (Office HCS 2014).

### National Health Interview Survey (2003)

This survey was to collect detailed information on the socio-economic status, self-reported health status, and health behaviour of the non-institutionalized Hungarian adult population. Two-stage random sampling was applied using the Central Data Processing, Registration and Election Office’s registry. The first step was the selection of communities (cities, towns and villages) where sampling probabilities were defined by population size. As second step, individuals were selected at random. Interviews were performed in the autumn of 2003 by field workers experienced in interview techniques and who had been trained before the survey for this specific interview study and the process of conducting the interview itself for minimizing the interviewers-related error. Of the planned 7000 interviews, 5072 were completed in the National Health Interview Survey. Data from the 4121 persons younger than 65 years were included in the analysis.

### National Health Interview Survey (2014)

This survey was designed in the same way as the National Health Interview Survey in 2003. The sampling and the interviewing was organized in the very same manner. The questionnaire applied was identical in the two data collection. This survey was supervised by EUROSTAT. The two-stage stratified random sample of 9431 was invited to participate in the survey. The number of completed questionnaires was 5826. Data of 4406 adults less than 65 years were utilized.

For the Roma surveys, the same instrument and sampling procedures were used in 2004 and in 2015. The target population lived in Roma settlements in North-eastern region of Hungary, where most Roma live. The Roma surveys collected data in Roma settlements because the misclassification bias from serious underreporting of Roma ethnicity experienced in the general population-based surveys was intended to be avoided. The data collection was carried out by Roma interviewers, supported by the local Roma self-government—by this way, the misclassification problem of self-identification could be minimized. Study protocols have been approved by the Ethical Committee of the Hungarian National Scientific Council on Health. (TUKEB 445/2003 and TUKEB 47062/2015) Informed consent was obtained from all individual participants included in the study.

### First Roma Health Survey (2004)

Before the health survey between 2001 and 2003, a detailed environmental survey was undertaken in three counties in which the Roma population is greatest; the researchers identified all such settlements (in which the population was almost exclusively Roma) and recorded the number of people living in them. In the environmental survey, settlements with at least four households were mapped, and the resulting plot was used as the basis for the health survey. The report on the environmental survey was published later. (Kosa et al. 2011). Collectively, approximately 62,000 persons lived in the Roma settlements in these counties (of a total population of the counties of 1877 243). A 2-stage sampling process was used: towns and villages in the three counties with identified settlements were selected randomly, and then households were selected with the random walk method, based on a map of the settlement. All adults, 18 years and older, in selected households were invited to participate. To maximise the response rate, interviewers were Roma and the survey was supported by local Roma civil society organizations. The target number was 1000 inhabitants of Roma settlements. Details have been reported previously (Kósa et al. 2007).

### Second Roma Health Survey (2015)

This survey (sampling, survey instrument) was exactly the same as the 2004 survey. Given the passage of time, the mapping of Roma settlements was rechecked by means of visits and the sampling frame was corrected. The resulting sampling frame contained 123 settlements where 52,099 people were living. The sample consisted of 1000 Roma adults.

### Questionnaire

We used internationally recommended survey tools when possible (Bruin et al. 1996; Eurostat 2014). Most questions were asked by the interviewer; some sensitive subjects such as alcohol consumption and discrimination were self-administered. Self-reported health was assessed with a standard 5-item question (Topp et al. 2015). We combined the categories “good” and “very good” as well as “bad” and “very bad” (and named them as “good or very good” and “bad or very bad”) to get useful statistical power for all analysed subsamples. Height and weight were self-reported, and body mass index (BMI) was calculated as body weight (kg) divided by height in meters squared (m^2^). BMI was categorized in accordance with WHO guidelines (WHO 1995).

The highest level of education, present employment status, living conditions, household income, and self-rated financial status were used to describe socio-economic status. The proportion of participants with only primary education, with bad or very bad perceived financial status, living in a one-room flat, with active employment, and mean household equivalent monthly income was calculated (total household income per month divided by the square root of the number of persons in the household).

Data on cigarette smoking, alcohol consumption, and social support were derived from answers given to multiple-response questions. Alcohol consumption was classified as heavy drinkers (more than seven standard drinks—a standard drink was defined as 12 g of pure alcohol—per week or more than three standard drinks on any day for women; more than 14 standard drinks per week or more than 5 standard drinks on any day for men), moderate drinker (weekly consumption but less than heavy drinking), occasional drinker (consumption less often than weekly), and abstinent (Vik et al. 2000). Smoking questions were from the European Health Interview Survey (EUROHIS) project. Within the regular smoker category, heavy and moderate smoking was classified as above and below 20 cigarettes a day. Former smoking was defined as at least 1 year of abstinence. Frequency (daily, weekly, less than weekly) of fruit and vegetable consumption, and preference for vegetable oil or lard were also included.

Self-reported frequency of health service use was collected (contacts with family physicians, dentists, and specialists from secondary care in the previous 12 months). Cervical and breast cancer screening were in the previous 5 and 2 years, respectively. Consumption of statins or fibrates was measured by self-report (Boruzs et al. 2016).

### Data analysis

Age- and sex-specific prevalence of measures were calculated. Subjects were categorized by age as follows: 18–29, 30–44, and 45–64 years (referred as younger, middle aged, older). The exact 95% confidence interval was computed for all point estimates. Stata 10.1 was used for statistical analyses. Differences in prevalences were evaluated by 95% confidence intervals presented. Changes between 2004 and 2015 were estimated as risk ratios, with 2004 serving as the reference. The age- and sex- specific prevalences, and risk ratios for almost all indicators were available for the general Hungarian population (detailed analysis for 2003, primary estimates for 2014), as well.

## Results

969 interviews were completed successfully in the Roma Health Behaviour Survey 2004 (96.9% response rate), and 905 interviews in the Roma Health Behaviour Survey, 2015 (90.5% response rate). Because the representation of people over 65 years was 3.4% (*n* = 33) in the Roma sample in the First Roma Health Survey, and 4.3% (*n* = 39) in the Second Roma Health Survey, the size of the strata of 65-X was too small to make reliable conclusions for this subgroup of the population, data from persons younger than 65 years (936 and 866, respectively) were included in the analysis.

### Socioeconomic status

Summary data are shown in Table 1. In both surveys, an extremely high proportion of adults with only primary education could be identified, which did not differ significantly. Between the two surveys, the share of active workers and the mean household income increased markedly in all age groups. The proportion of Roma living in one-room flats was high in both surveys and did not change. The proportion reporting bad or very bad self-assessed financial status decreased significantly in all age groups.

**Table 1:** Socioeconomic and self-perceived health status of Roma living in segregated settlements of North-eastern region of Hungary in 2004 and in 2015 and of the general Hungarian population according to the national health interview surveys in 2003 and in 2014 with corresponding 95% confidence intervals, and the changes of indicator values by age groups

	First surveys (2003/2004)			Second surveys (2014/2015)			Changes (ratio^#^)		
	18–29 years	30–44 years	45–64 years	18–29 years	30–44 years	45–64 years	18–29 years	30–44 years	45–64 years
Only primary education (%)									
Roma	73 (68, 77)	80 (75, 84)	87 (82, 91)	72 (67, 77)	84 (79, 88)	95 (91, 97)	0.99	1.05	1.09
General	16 (14, 19)	17 (15, 20)	29 (26, 32)	20 (20.2, 20.3)	10.77 (10.73, 10.81)	16.51 (16.46, 16.55)	1.25*	0.65*	0.59*
Active worker (%)									
Roma	17 (14, 21)	22 (18, 27)	11 (7, 16)	47 (41, 53)	54 (48, 60)	46 (39, 53)	2.76*	2.45*	4.18*
General	62 (59, 65)	79 (76, 81)	53 (51, 56)	50.4 (50.3, 50.5)	80.57 (80.52, 80.62)	61.35 (61.29, 61.41)	0.81*	1.03	1.15*
Mean household equivalent income per month, EUR									
Roma	170 (160, 180)	161 (153, 169)	159 (150, 168)	192 (182, 203)	185 (176, 193)	188 (165, 211)	1.13*	1.15*	1.18
General	332 (316, 348)	307 (293, 321)	300 (290, 309)	nd	nd	nd	–	–	–
Self-assessed financial status very bad or bad (%)									
Roma	59 (54, 64)	62 (56, 67)	65 (58, 71)	38 (32, 44)	38 (32, 44)	39 (32, 46)	0.64*	0.61*	0.60*
General	2 (1,5, 3)	4 (3, 5)	5 (4, 7)	14.41 (14.35, 14.47)	16.06 (16.01, 16.11)	18.57 (18.52, 18.62)	7.00*	4.00*	3.80*
Living in a one-room flat (%)									
Roma	11 (8, 14)	12 (9, 16)	15 (10, 20)	13 (10, 17)	13 (10, 17)	13 (10, 19)	1.18	1.08	1.27
General	9 (7, 11)	8 (6, 10)	6 (5, 8)	nd	nd	nd	–	–	–
Self-reported health status bad or very bad (%)									
Roma	3 (2, 5)	18 (14, 23)	50 (44, 57)	17 (13, 22)	20 (16, 25)	22 (17, 28)	5.67*	1.11	0.44*
General	4 (2, 6)	8 (7, 10)	25 (23, 27)	0.93 (0.92, 0.95)	2.82 (2.80, 2.84)	13.62 (13.58, 13.66)	0.23*	0.25*	0.52*
Self-reported health status good or very good (%)									
Roma	79 (75, 83)	48 (42, 55)	13 (9, 17)	46 (40, 52)	39 (33, 45)	26 (20, 33)	0.58*	0.81	2.00*
General	80 (75, 83)	52 (49, 56)	20 (19, 23)	90.76 (90.71, 90.80)	80.15 (80.10, 80.20)	51.19 (51.13, 51.25)	1.13*	1.54*	2.55*
Could do much or much more for own health (%)									
Roma	68 (64, 73)	53 (48, 59)	39 (33, 46)	55 (49, 61)	49 (43, 55)	47 (40, 55)	0.81*	0.92	1.21
General	88 (86, 90)	80 (77, 82)	66 (64, 69)	92.02 (91.98, 92.07)	87.76 (87.72, 87.80)	76.62 (76.57, 76.67)	1.05*	1.09*	1.15*
Discrimination in health care (%)									
Roma	27 (21, 33)	33 (26, 41)	47 (40, 55)	35 (31, 39)	15 (11, 19)	24 (18, 31)	1.30	0.42*	0.49*
General	nd	nd	nd	nd	nd	nd	–	–	–

*nd* no data

*Significant change

^#^Reference year 2004 for Roma survey and 2003 for Hungarian survey

### Self-rated health status and access to health care

There were divergent trends in self-perceived health status by age; significantly worse health was reported by younger Roma while older Roma reported significantly improved health. The proportion of persons believing that they could do much or very much to improve their health decreased among younger Roma but did not change in other age groups. What had been a very high proportion of responders who reported having experienced discrimination in encounters with health care in 2004 fell considerably among middle aged and older Roma, but was unchanged among younger Roma (Table 1).

### Health service use

During the period between the surveys, there was a significant increase in reported use of health services (any health service use in the previous 12 months) by both genders among younger and middle-aged Roma. However, this varied by the service used. Thus, Roma women at all ages, and the younger and middle-aged men visited the family physicians at the same rate in 2004 and 2015. Younger Roma men visited a family physician significantly more frequently in 2015 than in 2004. Use of secondary care and dental services were unchanged in Roma in all age–sex strata. Roma women reported fewer gynaecological investigations, significantly so among younger Roma. Reported breast cancer screening (mammography) among Roma women aged 45–64 years showed no significant difference. Concerning cholesterol-lowering therapy, 1.91% (95% confidence interval 1.14–3.01%) in 2004, and 5.31% (95% confidence interval 3.91–7.02%) in 2015 was the prevalence of regular users (Table 2).

**Table 2:** Health service utilization by Roma living in segregated settlements of North-eastern region of Hungary in 2004 and in 2015 and by the general Hungarian population according to the national health interview surveys in 2003 and in 2014 with corresponding 95% confidence intervals, and the changes of indicator values by sex and age groups

	First surveys (2003/2004)			Second surveys (2014/2015)			Changes (ratio^#^)		
	18–29 years	30–44 years	45–64 years	18–29 years	30–44 years	45–64 years	18–29 years	30–44 years	45–64 years
Women									
Use of any service in the previous 12 months									
Roma	65 (58, 71)	62 (55, 70)	78 (69, 85)	85 (79, 90)	85 (79, 91)	90 (82, 95)	1.31*	1.37*	1.15
General	74 (70, 77)	73 (69, 76)	80 (78,83)	nd	nd	nd	–	–	–
Contact with family physician in the previous 12 months									
Roma	70 (64, 76)	71 (63, 77)	86 (78, 91)	74 (67, 81)	69 (61, 76)	83 (74, 90)	1.06	0.97	0.97
General	62 (59, 65)	62 (59, 65)	77 (75, 79)	73.74 (73.63, 73.84)	73.2 (73.1, 73.3)	82.5 (82.4, 82.6)	1.19*	1.18*	1.06*
Consulting a specialist in the previous 12 months									
Roma	51 (44, 58)	42 (35, 50)	58 (49, 67)	47 (39, 56)	44 (36, 52)	50 (40, 60)	0.92	1.05	0.86
General	68 (64, 72)	68 (64, 71)	69 (66, 72)	64.1 (63.9, 64.2)	68.3 (68.2, 68.4)	69.6 (69.5, 69.7)	0.94	1.00	1.00
Use of dental service in the previous 12 months									
Roma	44 (38, 51)	33 (26, 41)	32 (24, 41)	33 (26, 41)	28 (21, 36)	17 (10, 26)	0.75	0.85	0.53
General	61 (57, 65)	50 (46, 54)	39 (36, 43)	62.8 (62.7, 63)	61.1 (61, 61.2)	46 (45.9, 46)	1.02	1.22*	1.18*
Gynaecologist appointment in the previous 5 years									
Roma	90 (85, 93)	82 (75, 87)	62 (53, 71)	76 (69, 82)	75 (67, 81)	55 (45, 65)	0.84*	0.91	0.89
General	90 (87, 92)	92 (89, 94)	86 (84, 87)	nd	nd	nd	–	–	–
Mammography in the previous 2 years									
Roma			25 (18, 34)			14 (8, 23)			0.56
General			70 (67, 74)			66.4 (66.3, 66.5)			0.94*
Cholesterol-lowering medication									
Roma	0 (0, 1.8)	1.2 (0.2, 4.4)	5.4 (2.0, 11.3)	6.5 (3.3, 11.4)	4.4 (1.8, 8.9)	11.5 (6.1, 19.3)	nc*	3.59	2.15
General	nd	nd	nd	nd	nd	nd	–	–	–
Men									
Use of any services in the previous 12 months									
Roma	42 (35, 49)	48 (40, 56)	67 (57, 75)	86 (80, 91)	86 (78, 90)	83 (74, 90)	2.05*	1.79	1.24
General	60 (56, 64)	61 (57,65)	72 (86, 75)	nd	nd	nd	–	–	–
Contact with family physician in the previous 12 months									
Roma	48 (41, 55)	51 (43, 59)	74 (65, 81)	66 (58, 74)	55 (46, 64)	67 (57, 76)	1.38*	1.08	0.91
General	67 (63, 71)	68 (64, 71)	73 (71, 75)	61 (60.9, 61.1)	62.75 (62.66, 62.84)	75.54 (75.47, 75.62)	0.91*	0.93*	1.04*
Consulting a specialist in the previous 12 months									
Roma	22 (16, 28)	23 (17, 31)	42 (34, 52)	34 (27, 42)	26 (19, 34)	37 (28, 47)	1.55	1.13	0.88
General	38 (33, 42)	35 (31, 38)	51 (47, 54)	41.62 (41.50, 41.73)	48.95 (48.86, 49.05)	59.22 (59.13, 59.30)	1.11	1.40*	1.16*
Use of dental service in the previous 12 months									
Roma	38 (31, 45)	26 (20, 34)	21 (14, 29)	28 (21, 36)	19 (12, 26)	17 (10, 26)	0.74	0.73	0.81
General	42 (37, 46)	40 (36, 44)	30 (27, 34)	53.83 (53.72, 53.95)	47.06 (46.96, 47.15)	39.67 (39.59, 39.76)	1.29*	1.18*	1.33*
Cholesterol-lowering medication									
Roma	0.5 (0, 2.8)	2.1 (0.4, 6.0)	5.2 (1.9, 11)	1.3 (0.2, 4.4)	4.4 (1.8, 8.8)	6.1 (2.5, 12.2)	2.47	2.1	1.77
General	nd	nd	nd	nd	nd	nd	–	–	–

*nc* not computable, *nd* no data

*Significant change

^#^Reference year 2004 for Roma survey and 2003 for Hungarian survey

### Health determinants

More Roma reported never smoking in all strata, other than older women; the increase was especially large among both men and women by 2015. The decreasing intensity of smoking among regular smokers could be detected in both sexes: the decrease of heavy smoking in both sexes among middle aged, and the increase of moderate smoking among middle-aged and older Roma women proved to be significant (Tables 3, 4).

**Table 3:** Prevalence of major risk factors among Roma women living in segregated settlements of North-eastern region of Hungary in 2004 and in 2015 and in the general Hungarian population according to the national health interview surveys in 2003 and in 2014 with corresponding 95% confidence intervals, and the changes of indicator values by age groups

	First surveys (2003/2004)			Second surveys (2014/2015)			Changes (ratio^#^)		
	18–29 years	30–44 years	45–64 years	18–29 years	30–44 years	45–64 years	18–29 years	30–44 years	45–64 years
Smoking									
Never									
Roma	40 (34, 47)	22 (17, 29)	32 (24, 41)	47 (39, 55)	41 (33, 50)	29 (20, 39)	1.18	1.86*	0.91
General	53 (49, 57)	47 (42, 51)	56 (53, 59)	61.2 (61.1, 61.3)	60.17 (60.08, 60.26)	50.77 (50.68, 50.85)	1.15*	1.28*	0.89*
Former									
Roma	9 (6, 14)	12 (7.6, 18)	13 (7.6, 20)	0 (0, 2)	2 (0, 6)	3 (1, 9)	0.00*	0.17*	0.23
General	13 (9, 19)	12 (9, 14)	16 (14, 19)	9.32 (9.25, 9.39)	15.35 (15.29, 15.42)	20.02 (19.95, 20.09)	0.69	1.25*	1.25*
Moderate									
Roma	25 (20, 32)	17 (11, 23)	12 (6, 19)	31 (23, 38)	33 (25, 41)	34 (25, 45)	1.24	1.94*	2.83*
General	24 (21, 27)	24 (21, 28)	19 (17, 22)	3.6 (3.56, 3.64)	2.11 (2.09, 2.14)	0.7 (0.68, 0.71)	0.13*	0.08*	0.04*
Heavy									
Roma	25 (20, 32)	49 (41, 57)	44 (34, 53)	23 (16, 30)	24 (18, 32)	33 (24, 44)	0.92	0.49*	0.75
General	12 (10, 15)	18 (15, 21)	9 (7, 10)	25.88 (25.78, 25.98)	22.36 (22.29, 22.44)	28.52 (28.44, 28.59)	2.08*	1.22*	3.11*
Alcohol consumption									
Abstinent									
Roma	83 (77, 88)	82 (76, 88)	91 (84, 95)	71 (56, 84)	69 (58, 80)	62 (46, 76)	0.86	0.84	0.68*
General	57 (53, 61)	55 (51, 59)	61 (57, 65)	30.91 (30.81, 31.02)	36.52 (36.43, 36.61)	34.37 (34.29, 34.45)	0.53*	0.65*	0.56*
Occasional									
Roma	14 (10, 20)	13 (8, 19)	7 (4, 14)	20 (10, 35)	20 (11, 32)	24 (12, 40)	1.43	1.54	3.43
General	36 (32, 40)	35 (31, 39)	26 (23, 29)	60.68 (60.57, 60.79)	54.63 (54.54, 54.73)	53.6 (53.52, 53.69)	1.67*	1.54*	2.04*
Moderate									
Roma	2 (1, 5)	3 (1, 7)	1 (0, 6)	0 (0, 8)	2 (0, 9)	2 (0, 13)	0.00	0.67	2.00
General	4 (3, 7)	8 (6, 10)	11 (9, 13)	7.63 (7.57, 7.69)	8.33 (8.28, 8.38)	9.91 (9.86, 9.96)	1.75*	1.00	0.82
Heavy									
Roma	1 (0.2, 4)	2 (0, 6)	1 (0, 6)	9 (3, 21)	10 (4, 20)	12 (4, 26)	9.00	5.00	12.00
General	3 (2, 5)	2 (1, 4)	2 (2, 4)	0.78 (0.75, 0.8)	0.52 (0.51, 0.53)	2.11 (2.09, 2.13)	0.23*	0.25*	1.00
BMI									
Overweight									
Roma	14 (10, 20)	28 (22, 36)	34 (26, 44)	28 (21, 36)	34 (26, 42)	27 (19, 37)	2.00*	1.21	0.79
General	14 (12, 17)	24 (22, 29)	38 (35, 41)	12.83 (12.76, 12.91)	21.37 (21.3, 21.45)	34.59 (34.51, 34.67)	0.86	0.88*	0.89*
Obese									
Roma	4 (2, 8)	11 (7, 17)	21 (15, 30)	27 (20, 34)	22 (16, 30)	23 (15, 33)	6.75*	2.00*	1.10
General	5 (4, 8)	14 (12, 17)	25 (22, 28)	6.47 (6.41, 6.53)	15.03 (14.96, 15.09)	26.53 (26.45, 26.6)	1.20	1.07	1.04
Diet									
Use of vegetable oils									
Roma	26 (21, 33)	30 (23, 37)	28 (21, 37)	68 (60, 75)	67 (59, 75)	59 (48, 69)	2.62*	2.23*	2.11*
General	70 (66, 73)	63 (59 ,67)	61 (57, 64)	nd	nd	nd	–	–	–
Consumption of fruit and vegetables daily									
Roma	32 (26, 39)	36 (29, 44)	36 (27, 45)	60 (52, 68)	58 (50, 66)	48 (38, 58)	1.88*	1.61	1.33
General	55 (50, 59)	66 (63, 70)	77 (74, 79)	61.79 (61.67, 61.9)	68.34 (68.25, 68.42)	73.75 (73.67, 73.82)	1.11*	1.03	0.95*

*nd* no data

*Significant change

^#^Reference year 2004 for Roma survey and 2003 for Hungarian survey

**Table 4:** Prevalence of major risk factors among Roma men living in segregated settlements of North-eastern region of Hungary in 2004 and in 2015 and in the general Hungarian population according to the national health interview surveys in 2003 and in 2014 with corresponding 95% confidence intervals, and the changes of indicator values by age groups

	First surveys (2003/2004)			Second surveys (2014/2015)			Changes (ratio^#^)		
	18–29 years	30–44 years	45–64 years	18–29 years	30–44 years	45–64 years	18–29 years	30–44 years	45–64 years
Smoking									
Never									
Roma	31 (25, 38)	20 (15, 28)	13 (8, 20)	44 (36, 52)	37 (29, 46)	28 (19, 37)	1.42	1.85*	2.15
General	41 (37, 44)	38 (34, 42)	29 (26, 32)	50.23 (50.11, 50.34)	45.07 (44.97, 45.16)	37.66 (37.57, 37.74)	1.22*	1.18*	1.28*
Former									
Roma	5 (3, 9)	6 (3, 11)	17 (11, 25)	1 (0, 5)	0,7 (0, 4)	6 (2, 12)	0.20	0.12	0.35
General	8 (6, 11)	17 (14, 20)	32 (28, 35)	7.57 (7.51, 7.63)	16.03 (15.97, 16.1)	29.57 (29.49, 29.65)	0.88	0.94	0.91
Moderate									
Roma	14 (9, 19)	13 (8, 19)	17 (11, 25)	21 (14, 28)	25 (18, 33)	32 (24, 42)	1.50	1.92	1.88
General	28 (25, 32)	15 (12, 19)	15 (13, 18)	3.08 (3.04, 3.12)	2.04 (2.01, 2.06)	1.87 (1.84, 1.89)	0.11*	0.13*	0.12*
Heavy									
Roma	50 (43, 57)	61 (53, 69)	53 (43, 63)	34 (27, 43)	38 (29, 46)	34 (25, 44)	0.68	0.62*	0.64
General	23 (20, 27)	30 (26, 35)	24 (22, 28)	39.12 (39, 39.23)	36.86 (36.77, 36.95)	30.9 (30.82, 30.98)	1.70*	1.20*	1.25*
Alcohol consumption									
Abstinent									
Roma	44 (37, 51)	45 (37, 53)	59 (50, 68)	46 (34, 58)	40 (30, 51)	42 (30, 55)	1.05	0.89	0.71
General	30 (26, 33)	25 (21, 29)	23 (20, 27)	14.51 (14.43, 14.59)	17.47 (17.39, 17.54)	15.95 (15.89, 16.01)	0.47*	0.68*	0.65*
Occasional									
Roma	16 (11, 22)	14 (9, 21)	12 (7, 20)	26 (16, 37)	26 (17, 37)	17 (9, 28)	1.63	1.86	1.42
General	36 (32, 40)	26 (22, 30)	17 (14, 20)	56.67 (56.56, 56.78)	49.5 (49.41, 49.59)	37.78 (37.69, 37.86)	1.56	1.88*	2.18*
Moderate									
Roma	24 (19, 31)	26 (19, 33)	16 (10, 24)	0 (0, 5)	6 (2, 13)	6 (2, 14)	0.00*	0.23*	0.38
General	23 (20, 26)	31 (27, 35)	39 (35, 42)	21.9 (21.81, 21.99)	26.4 (26.32, 26.48)	33.34 (33.26, 33.43)	0.91*	0.84*	0.85*
Heavy									
Roma	16 (12, 22)	16 (11, 23)	13 (8, 21)	28 (19, 40)	28 (19, 39)	35 (24, 47)	1.75	1.75	2.69*
General	12 (9, 15)	18 (15, 22)	21 (18, 25)	6.92 (6.87, 6.98)	6.63 (6.58, 6.68)	12.93 (12.87, 12.99)	0.58	0.37*	0.57*
BMI									
Overweight									
Roma	37 (30, 44)	40 (32, 48)	36 (28, 46)	29 (22, 37)	40 (32, 49)	29 (20, 38)	0.78	1.00	0.81
General	26 (23, 30)	43 (39, 48)	44 (40, 48)	25.36 (25.26, 25.46)	41.96 (41.87, 42.06)	44.36 (44.27, 44.44)	0.96	0.95	1.00
Obese									
Roma	5 (3, 9)	18 (13, 26)	22 (15, 31)	24 (17, 31)	20 (14, 27)	21 (14, 30)	4.80*	1.11	0.95
General	10 (8, 13)	19 (15, 23)	24 (21, 27)	9.27 (9.21, 9.34)	18.6 (18.53, 18.67)	30.89 (30.81, 30.97)	0.90	0.95	1.25*
Diet									
Use of vegetable oils									
Roma	21 (16, 27)	28 (21, 36)	22 (16, 31)	58 (50, 66)	50 (41, 59)	57 (47, 67)	2.76*	1.79*	2.59*
General	61 (56, 66)	62 (58, 66)	59 (56, 61)	nd	nd	nd	–	–	–
Consumption of fruit and vegetables daily									
Roma	28 (22, 35)	31 (24, 39)	37 (28, 46)	39 (31, 48)	41 (32, 50)	33 (24, 43)	1.39	1.32	0.89
General	42 (38, 46)	49 (45, 54)	60 (56, 63)	56.63 (56.52, 56.74)	55.46 (55.37, 55.55)	61.93 (61.84, 62.01)	1.33*	1.12	1.02

*nc* not computable, *nd* no data

*Significant change

^#^Reference year 2004 for Roma survey and 2003 for Hungarian survey

The proportion of older women who were abstinent remained high but decreased significantly. There was no change in rates of abstinence among women at other ages and among men of all ages. Among men, there was a shift from moderate to heavy drinking at all ages, reaching significance among those who were younger and middle aged. The distribution of BMI worsened among younger Roma (in both sexes) between 2004 and 2015, with obesity becoming significantly more frequent. The same trend was observed among middle-aged women. The use of vegetable oil for cooking by everyone became much more common in the last decade. Among women, there was a significant improvement in daily consumption of fruits and vegetables, reaching significance among the younger and older groups. Among men, a similar trend could be detected but only among the younger (Tables 3, 4).

Compared with the general Hungarian population, the gap narrowed in relation to employment, perceived financial status, use of primary care, smoking, and daily consumption of fruits and vegetables. However, the gap with the general population widened for education, self-reported health status and positive attitudes to health among younger people, use of secondary and dental care, overweight, and alcohol consumption, due to the worsening situation among Roma.

## Discussion

This study suffers from one inevitable limitation. Namely, concerning the extreme heterogeneity of Roma and the given definitional problems that we discussed in details previously (Kósa et al. 2007), it is very difficult—if not impossible—to undertake a truly representative survey of Roma. Our approach captures those Roma who are most disadvantaged, and in case of their proper collaboration are the most identifiable. This approach has been employed in previous surveys of Roma health and living conditions (Kosa et al. 2011; Kósa et al. 2007) and using exactly the same survey methods (preventing the influence of social desirability bias on Roma to non-Roma comparisons), the data sets of health behaviour surveys should be comparable. Unfortunately, there were no health interview surveys in Hungary which could provide results for the general population living in the same region which were covered by the Roma surveys. Consequently, the changes in general population calculated in the analysis are only approximations of the changes in general population representing the same geographical area that was covered by the Roma surveys.

Our analysis does, however, have some important advantages. First, it uses an innovative approach to identify the most vulnerable elements of the Roma population. Second, it avoids bias that may arise from differences in self-identification by identifying the target group on the basis of the observable characteristics of where they live. Third, it achieved a very high response rate in both waves, which we attribute to the highly participatory nature of the research, involving representatives of the Roma population at all stages (design, conduct, and interpretation of the study).

The findings reveal a number of changes, for better and for worse. On one hand, Roma continue to experience severe disadvantage when judged by the educational status, and living conditions; moreover, use of some preventive services, such as mammography and gynaecological screening, actually declined. Alcohol consumption also showed deterioration, as did self-rated health status (except for older Roma), BMI and the belief that one can act to improve one’s health. On the other hand, employment and income improved, perceived discrimination decreased markedly, use of many health services increased, as did uptake of cholesterol lowering drug consumption. There were also improvements in smoking and diet.

While noting the problem of attribution, many of these changes can plausibly be linked to government policies over the past decade, although few are specifically targeted at Roma. For example, the Hungarian government set out its priorities, accompanied by a list of short-term actions, in the “Semmelweis Plan for the Rescue of Health Care” in 2010.

One factor is likely to be the decision by the Hungarian government to quadruple the budget for public works between 2010 and 2015 provided for all Hungarian municipalities. This is especially relevant for villages in the North-eastern region of Hungary where segregated Roma settlements are concentrated. The majority of workers participating in the programme are from deprived Roma communities. This provides a monthly salary of approximately 162 USD per month, which is the only regular income for many Roma.

The decline in smoking in the last decade coincides with the implementation of a multifaceted anti-tobacco programme. Smoking has been banned in public and work places, and there have been major restrictions on marketing, with sales restricted to designated outlets, and restricted to those 18 and over. The Government has also steadily increased taxation so that the price/pack of the most popular price category (MPPC) was 0.60 USD in 1992, 2.13 USD in 2003, and about 3.54 USD in 2013. Allowing for inflation, this is a more than doubling of the price. It is likely that rates will decline further; in 2015, Hungary introduced a new tax with steeply progressive rates on tobacco products (varying between 0.2–4.5%, depending on the companies’ turnover), referred to as a “health contribution”.

In contrast, the worsening pattern of alcohol consumption has coincided with a liberalization of laws on alcohol distillation in 2010. This was previously tightly regulated and taxed, with production only permitted by licensed distilleries. Black-market production of palinka (local brandy) was always widespread in the Hungarian countryside, where the majority of Roma live, but it was encouraged by new rules allowing distillation of 86 litres palinka a year at home free from tax. It is estimated that more than 15 million litres of palinka/year has been distilled at home. Because of the tax exemption, its price is relatively low, increasing its availability. This income appreciation is relatively stronger among the rather poor Roma adults, who do not distil at home spirits traditionally, not possessing the required minimal knowledge and equipment.

Lard was traditionally used for cooking in Hungary but it has progressively been displaced by cooking oil, which overtook it in 2000. This has been supported by the pricing policy: in 2009, the price of lard increased by 30.7%, but the price of cooking oil decreased 15.7%. The marked increase in daily fruit and vegetable consumption between survey periods also coincides with an active governmental policy. A new tax on “unhealthy” food, i.e. products such as soft drinks, energy drinks, pre-packed sweetened products, salty snacks and condiments, was introduced in Hungary in 2011, in close cooperation with WHO Europe. Hungary was among the first countries in Europe where such taxes were imposed. A report by the European Commission concluded that these “fat taxes” have worked, contributing to a reduction of salt, sugar and fat consumption (European Commission 2014a).

Although, the study design and the data processing do not allow drawing causal conclusions, these data suggest that changes in Roma living conditions and health can plausibly be linked to government policies, but they continue to experience severe disadvantage.

Although Hungary participated in the Decade of Roma Inclusion, despite improvements in employment of Roma, the lack of any significant change in living conditions and education, coupled with the worsening of certain health-related behaviours suggest that consistent with evidence from other sectors (Jovanovic 2015; Rostas et al. 2012), its achievements have been limited.

There is now an extensive body of research showing that Roma ethnicity has consequences for health beyond the low education and income and adverse living conditions of Roma (Balazs et al. 2014; Foldes et al. 2012; Kosa et al. 2015; Marmot 2013; Paulik et al. 2011; Voko et al. 2009). However, the lack of a robust surveillance system makes it impossible to assess progress in Decade of Roma Inclusion (Adany 2014). The secretariat for the Decade of Roma Inclusion, together with the EU Fundamental Rights Agency Working Party on Roma, has created a set of Indicators of integration of Roma and established the basic set of indicators. However, only three relate to health. In the “Framework for National Roma Integration Strategies up to 2020”, the European Commission has argued for a targeted approach to the different components of Decade of Roma Inclusion. However, a major obstacle has been the inability to define the target population (European Commission 2013, 2014b, 2015).

### Conclusions

Our experience suggests that, while recognising that it captures preferentially the most disadvantaged parts of the Roma population, the use of Roma settlements could be a pragmatic approach to monitoring, especially now that we have shown that this is reproducible over a decade, and suitable for monitoring changes in health inequalities in connection with policy initiatives, and especially the effects of targeted interventions.

## Electronic supplementary material

Below is the link to the electronic supplementary material.

Supplementary material 1 (DOCX 21 KB)
