# Supplementary material for: The decade of Roma Inclusion: did it make a difference to health and use of health care services?
Source: Int J Public Health. 2017 Mar 29;62(7):803–15. doi: 10.1007/s00038-017-0954-9 (PMC5585300; doi:10.1007/s00038-017-0954-9)
Supplement: Supplementary file 1 — Supplementary material 1 (DOCX 21 KB) [file 38_2017_954_MOESM1_ESM.docx]

Questions from the survey questionnaire utilized in preparation of the paper

**Socio-demographic status**

a) Sex

1. male

2. female

b) Age

[year]:

c) Highest level of education completed (Educational attainment)

1. pre-primary education

2. primary education

3. vocational education without graduation

4. vocational education with graduation

5. high school with graduation

6. tertiary education; bachelor level or equivalent

7. tertiary education; master level or equivalent

8. I don’t know

9. no answer

d) Do you carry out a job (self-declared labor status)?

1. yes

2. no

3. I don’t know

4. no answer

e) Net monthly income of the household

1. Hungarian forint:

2. I don’t know

3. no answer

f) How is your financial status? Is it…

1. very good

2. good

3. fair

4. bad

5. very bad

6. I don’t know

7. no answer

g) What is the number of rooms in your house or flat not taking into consideration kitchen bathroom, and anteroom?

1. [number of rooms]:

2. I don’t know

3. no answer

**Self-rated general health**

a) Thinking on last 12 months, how is your health in general? Is it…

1. very good

2. good

3. fair

4. bad

5. very bad

6. I don’t know

7. no answer

b) How much do you think you can do for your health?

1. very much

2. much

3. not much

4. nothing

5. I don’t know

6. no answer

**Life style**

a) How tall are you without shoes?

1. [cm]:

2. I don’t know

3. no answer

b) How much do you weigh without clothes and shoes?

1. [kg]:

2. I don’t know

3. no answer

c) Thinking on last four weeks, how often did you eat fresh fruits or vegetables?

1. more than once a day

2. once a day

3. once every second or third days

4. once a week

5. less than once a week

6. never

7. I don’t know

8. no answer

d) What kind of fat do you prefer at home cooking?

1. vegetable oil

2. lard

3. vegetable oil and lard

4. butter

5. margarine

6. neither of them

7. never cook at home

8. I don’t know

9. no answer

e) Do you smoke?

1. yes, daily

2. yes, occasionally, a few times a week

3. yes, occasionally, a few times a month

4. yes, occasionally, less often than a month

5. no, ceased

6. no, never

7. I don’t know

8. no answer

f) On average, how many cigarettes do you smoke each day?

1. [number of cigarettes]:

2. I don’t know

3. no answer

g) In the past 12 months, how often have you had an alcoholic drink of any kind [beer, wine, cider, spirits, cocktails, premixes, liquor, homemade alcohol…]?

1. every day or almost every day

2. 3-4 days a week

3. 1-2 days a week

4. 1-3 days in a month

5. Less than once a month

6. not in the past 12 months, as I no longer drink alcohol

7. I don’t know

8. no answer

h) Thinking of last week, how much alcohol containing beverages did you drink?

h1) on Monday

h11) beer

1 [dl]:

2. I don’t know

3. no answer

h12) wine or campaign

1 [dl]:

2. I don’t know

3. no answer

h13) vermouth, port wine, sherry

1 [dl]:

2. I don’t know

3. no answer

h14) spirit

1 [dl]:

2. I don’t know

3. no answer

h2) on Tuesday

h21) beer

1 [dl]:

2. I don’t know

3. no answer

h22) wine or campaign

1 [dl]:

2. I don’t know

3. no answer

h23) vermouth, port wine, sherry

1 [dl]:

2. I don’t know

3. no answer

h24) spirit

1 [dl]:

2. I don’t know

3. no answer

h3) on Wednesday

h31) beer

1 [dl]:

2. I don’t know

3. no answer

h32) wine or campaign

1 [dl]:

2. I don’t know

3. no answer

h33) vermouth, port wine, sherry

1 [dl]:

2. I don’t know

3. no answer

h34) spirit

1 [dl]:

2. I don’t know

3. no answer

h4) on Thursday

h41) beer

1 [dl]:

2. I don’t know

3. no answer

h42) wine or campaign

1 [dl]:

2. I don’t know

3. no answer

h43) vermouth, port wine, sherry

1 [dl]:

2. I don’t know

3. no answer

h44) spirit

1 [dl]:

2. I don’t know

3. no answer

h5) on Friday

h51) beer

1 [dl]:

2. I don’t know

3. no answer

h52) wine or campaign

1 [dl]:

2. I don’t know

3. no answer

h53) vermouth, port wine, sherry

1 [dl]:

2. I don’t know

3. no answer

h54) spirit

1 [dl]:

2. I don’t know

3. no answer

h6) on Saturday

h61) beer

1 [dl]:

2. I don’t know

3. no answer

h62) wine or campaign

1 [dl]:

2. I don’t know

3. no answer

h63) vermouth, port wine, sherry

1 [dl]:

2. I don’t know

3. no answer

h64) spirit

1 [dl]:

2. I don’t know

3. no answer

h7) on Sunday

h71) beer

1 [dl]:

2. I don’t know

3. no answer

h72) wine or campaign

1 [dl]:

2. I don’t know

3. no answer

h73) vermouth, port wine, sherry

1 [dl]:

2. I don’t know

3. no answer

h74) spirit

1 [dl]:

2. I don’t know

3. no answer

**Health care usage**

a) During the past 12 months, how many times did you consult a health professional on your own behalf?

1. [number of times]:

2. I don’t know

3. no answer

b) During the past 12 months, how many times did you consult a family physician on your own behalf?

1. [number of times]:

2. I don’t know

3. no answer

c) During the past 12 months, how many times did you consult a specialist on your own behalf?

1. [number of times]:

2. I don’t know

3. no answer

d) During the past 12 months, how many times did you consult a dentist on your own behalf?

1. [number of times]:

2. I don’t know

3. no answer

e) When was the last time you had a mammography (breast X-ray)?

1. within the past 12 months

2. 1 to less than 2 years

3. 2 years or more

4. never

5. I don’t know

6. no answer

f) When was the last time you had a gynecological appointment?

1. within the past 12 months

2. less than 3 years

2. less than 5 years

3. 5 years or more

4. never

5. I don’t know

6. no answer

g) Did your family physician prescribe cholesterol lowering drug for you?

1. yes

2. never

3. I don’t know

4. no answer

h) Do you take the cholesterol lowering drug as it is recommended?

1. yes

2. no

3. I don’t know

4. no answer

i) Have you experienced any personal discrimination in health care?

1. yes

2. no

3. I don’t know

4. no answer
